# HSV-1 Modulates IL-6 Receptor Expression on Human Dendritic Cells

**DOI:** 10.3389/fimmu.2020.01970

**Published:** 2020-08-26

**Authors:** Alexandra Birzer, Adalbert Krawczyk, Christina Draßner, Christine Kuhnt, Petra Mühl-Zürbes, Christiane Silke Heilingloh, Alexander Steinkasserer, Linda Popella

**Affiliations:** ^1^Department of Immune Modulation, Universitätsklinikum Erlangen, Erlangen, Germany; ^2^Institute for Virology, University Hospital Essen, University of Duisburg-Essen, Essen, Germany; ^3^Department of Infectious Diseases, University Hospital Essen, University of Duisburg-Essen, Essen, Germany

**Keywords:** IL-6 receptor, mature dendritic cells, HSV-1, bystander cells, L-particles, vhs

## Abstract

Dendritic cells (DCs) are the guardians of the immune system since they are located in the majority of peripheral tissues. In addition, they are crucial for the induction of an effective immune response based on their unique capacity to stimulate naive T cells. During co-evolution, the human pathogen herpes simplex virus type 1 (HSV-1) has evolved several immune evasion mechanisms in order to subvert the host's immune system especially by targeting DC biology and function. Here we demonstrate that HSV-1 infection influences the IL-6 receptor (IL6R) expression both on protein and mRNA levels in/on human monocyte-derived mature DCs (mDCs). Surprisingly, reduced IL6R expression levels were also observed on uninfected bystander mDCs. Mechanistically, we clearly show that HSV-1-derived non-infectious light (L-) particles are sufficient to trigger IL6R regulation on uninfected bystander mDCs. These L-particles lack the viral DNA-loaded capsid and are predominantly produced during infection of mDCs. Our results show that the deletion of the HSV-1 tegument protein vhs partially rescued the reduced IL6R surface expression levels on/in bystander mDCs. Using a neutralizing antibody, which perturbs the transfer of L-particles to bystander mDCs, was sufficient to rescue the modulation of IL6R surface expression on uninfected bystander mDCs. This study provides evidence that L-particles transfer specific viral proteins to uninfected bystander mDCs, thereby negatively interfering with their IL6R expression levels, however, to a lesser extend compared to H-particles. Due to their immune-modulatory capacity, L-particles represent an elaborated approach of HSV-1-mediated immune evasion.

## Introduction

Due to their unique ability to efficiently prime naive T cells, dendritic cells (DCs) are the most potent antigen presenting cells and thus play a crucial role for the induction of efficient adaptive immune responses (1). Immature DCs (iDCs) represent guardians of the immune system since they are present in the majority of all peripheral tissues, where they capture (foreign) antigens. There, iDCs are especially important to detect and take up foreign antigens facing the host's immune system. As a consequence, DCs undergo maturation, leading to fundamental changes in their surface expression pattern, such as upregulation of costimulatory molecules, e.g., cluster of differentiation (CD) 40, CD86, CD80, the functionally-important molecule CD83, major histocompatibility complex I and II (MHC class I and II) molecules or chemokine receptors, such as CCR7 and CXCR4 (1–3). The upregulation of CCR7 and CXCR4 empowers mature DCs (mDCs) to migrate along CCL19/CCL21 and CXCL12 chemokine gradients, respectively, into the draining lymph node via lymphatic vesicles (4). By contrast to iDCs, mDCs lose their high capacity for antigen uptake and gain the function to process and present antigens in the context of MHC molecules to naive T cells in draining lymph nodes, the major sites of antigen presentation (5). Due to these abilities, DCs act at the interface of the innate and adaptive immune system and are indispensable for the induction of an effective immune response. Therefore, it is not surprising that several pathogens have acquired strategies to hamper DC functions.

Herpes simplex virus type 1 (HSV-1) is a human pathogenic member of the α-herpesvirus subfamily. HSV-1 contains, common for all members of the herpesvirus family, a capsid protecting the viral DNA, the protein rich tegument layer and the envelope, which is equipped with several glycoproteins on the surface (6). HSV-1 is able to replicate in several host species, such as mice and monkeys (7–9), and different cell types, e.g., fibroblasts, epithelial cells or immune cells, such as DCs (10–12). While HSV-1 efficiently replicates in epithelial cells and thus produces two different particle types, mature heavy (H-) and non-infectious light (L-) particles, the viral replication in DCs is dependent on their maturation status. In iDCs, HSV-1 exploits cellular autophagy for nuclear lamin degradation, which facilitates the nuclear egress of viral capsids into the cytoplasm and thus the production of mature infectious virions (13). In sharp contrast, in mDCs HSV-1 is not able to accomplish its complete replication cycle since the degradation of nuclear lamins is inhibited by intrinsic blockade of autophagic turnover, thereby perturbing the generation of mature virions (13). Therefore, HSV-1-infected mDCs predominantly release non-infectious L-particles, which lack the capsid and thus the viral genome. Nevertheless, L-particles transport certain viral proteins to uninfected bystander DCs thereby hampering vital DC functions (12, 14–16).

HSV-1 has evolved several mechanisms to exploit the human immune system. In the past years, multiple immune escape mechanisms mediated by HSV-1 interfering with DC recognition, have been described. Notable examples are the impairment of proper T cell stimulation (17) and the degradation of CD83 (18). This functionally important surface molecule inhibits degradation of MHC class II molecules via blockage of the ubiquitin ligase MARCH1, thereby stabilizing MHC class II expression on DCs and consequently T cell stimulation (19, 20). In addition, HSV-1 also reduces the migratory capacity of mDC toward lymphoid tissue-specific chemokines, such as CCL19 or CXCL12, mediated by reduced chemokine receptor expression levels (21). Furthermore, HSV-1-infected mDCs exhibit a strongly increased cell adhesion, mediated via the upregulation of integrin activity, resulting from the virally-induced degradation of cytohesin-1 interacting protein [CYTIP (22)].

The IL-6 signaling pathway plays an important role in eliciting pro- as well as anti-inflammatory responses (23–25). The IL-6 receptor complex, which transduces IL-6-dependent signaling, is composed of the membrane-bound IL-6 receptor α (IL6R) and two components of its signal transducer glycoprotein 130 (gp130). While gp130 is ubiquitously expressed on all cells, IL6R expression is restricted to distinct cell types, such as hepatocytes and immune cells (26, 27). In the past years, immune cells expressing IL6R were extended to monocyte-derived DCs which also express the signal transducer gp130 (28, 29). In mDCs, IL6R protein is predominantly present intracellularly, however, the receptor is also expressed on the plasma membrane, where it cycles to intracellular compartments, such as endosomes or trans-Golgi (29). On cells expressing IL6R, e.g., DCs, the classical signaling pathway is induced via IL-6 binding to the IL6R (23, 30). In contrast, on cells lacking IL6R expression, the IL-6 signaling pathway can also be activated via the IL-6 interaction with a soluble form of IL6R (sIL6R), which dimerizes with cell surface-exposed gp130, and is therefore called trans-signaling (31). Cells producing the soluble variant of the IL6R are, e.g., T cells, DCs and cancer cells (28, 32, 33). Both arms of IL-6 signaling result in signal transducer and activator of transcription 3 (STAT3) phosphorylation followed by its nuclear translocation, which in turn leads to target gene activation mediating cell proliferation, differentiation or the induction of immune responses (34). The IL-6 signaling pathway is an important inductor of an anti-viral immune response (35, 36) and thus frequently targeted by several viruses, including Enterovirus 71 or influenza A virus (37, 38). However, the regulation of the signaling components, i.e., IL6R and STAT3, differs between distinct viruses and infected cell types (39, 40).

Within the present study, we analyze if and how HSV-1 targets the IL6R expression by mDCs and show that, compared to mock-infected control, mDCs express decreased IL6R levels on directly infected mDCs and in addition also on uninfected bystander mDCs. Furthermore, our investigations revealed a novel role for L-particles, since these non-infectious viral particles were sufficient to induce IL6R modulation on uninfected bystander mDCs, however, to a lesser extend as H-particles on directly HSV-1-infected mDCs. In this respect, we provide evidence that viral proteins are transferred via L-particles from HSV-1-infected mDCs to uninfected bystander mDCs, thereby negatively interfering with IL6R surface expression.

## Materials and Methods

### Amplification of Virus Strains

The strain HSV-1/syn 17^+^/CMV-EGFP/UL43 (CMV–cytomegalovirus, EGFP–enhanced green fluorescent protein, UL–unique long), herein designated as wildtype (wt), was generated from the laboratory strain HSV-1 syn 17^+^ (41) via insertion of a GFP expression cassette into the UL43 locus of the HSV-1 genome (BioVex). The GFP cassette is under the control of a CMV promoter and the ablation of UL43 is not obligatory for HSV-1 replication (42, 43). The HSV-1-GFPΔKan-UL41, herein designated as HSV-1 Δvhs, also possesses an EGFP expression cassette inserted into the UL41 gene locus encoding vhs (kindly provided by Martin Messerle, Hannover Medical School, Germany). The UL41 gene encodes for the viral virion host shutoff (vhs) protein which functions as viral endoribonuclease and degrades cellular and viral mRNAs (44, 45). For amplification of HSV-1 wt and HSV-1 Δvhs virus strains, 90% confluent BHK21 cells in 15 T_175_ cell culture flasks were washed once with PBS and HSV-1-infected in infection medium (RPMI 1640 (Lonza, Switzerland), 20 mM HEPES) supplemented with HSV-1 virions at a multiplicity of infection (MOI) of 0.01. After an infection period of 1 h on an orbital shaker at RT, 20 mL DMEM medium [supplemented with 10% FCS, 2 mM L-glutamine, 100 U/mL Penicillin, 100 U/mL Streptomycin and 1% non-essential amino acids (100× stock)] were added per cell culture flask and cells were subsequently incubated at 37°C and 5% CO_2_. Four days post infection, supernatants containing HSV-1 particles were separated from cell debris via centrifugation at 2,575 × g at 4°C for 10 min. Afterwards, supernatants were transferred into high speed centrifugation tubes and centrifuged at 39,742 × g at 4°C for 2 h. For resuspension of the virus pellet, pellets were overlaid with 150 μL MNT buffer for virus amplification (30 mM MES, 100 mM NaCl, 20 mM Tris) or 150 μL DMEM without phenol red (high glucose; Sigma-Aldrich, Germany) for particle isolation and stored at 4°C overnight. For the preparation of virus stocks, virus suspension was aliquoted into cryo-vials for storage at −80°C. For L-particle isolation, virus suspension was directly loaded onto a Ficoll gradient (see “Isolation of HSV-1-derived particles”). Virus titration was performed as previously described (46).

### Isolation of HSV-1-Derived Particles

H- and L-particles were isolated from supernatants derived from HSV-1-infected BHK21 cells as described in “Amplification of virus strains.” The isolation of H- and L-particles was performed according to a previously published protocol (47). Briefly, a gradient of 5 to 20% Ficoll PM 400 (Sigma-Aldrich, Germany) was loaded with the virus suspension and centrifuged at 26,000 × g for 2 h at 4°C. The H- and L-particle bands were harvested via punctuation with a needle, transferred into centrifugation tubes (Beckman Coulter, USA) and were filled up with 30 mL DMEM without phenol red (high glucose, Sigma-Aldrich, Germany). Both particle types were centrifuged at 80,000 × g for 2 h at 4°C. For further usage, particles were resuspended in an appropriate amount of DMEM without phenol red depending on the pellet size and stored at −80°C. In order to inactivate contaminating H-particles, the L-particle preparations were UV-irradiated three times applying 0.12 J/cm^2^ in a Vilber Luormat (Biometra, Germany).

### Generation of Human Monocyte-Derived Dendritic Cells (DCs)

Peripheral blood mononuclear cells (PBMCs) were derived from leukoreduction system chambers (LRSC) from different healthy donors (48). In brief, lymphoprep solution (Nycomed Pharma AS, Norway) was carefully overlaid with LRSC blood, diluted 1:5 in PBS supplemented with 10% ACD-A (Lonza, Switzerland), and the gradient was centrifuged at 400 × g at RT for 30 min. Afterwards, mononuclear cells were collected (intermediate phase) and washed three times with ice-cold PBS supplemented with 1 mM EDTA. Afterwards, PBMCs were resuspended in 10 mL RPMI 1640, centrifuged again at 300 × g for 5 min and incubated in 25 mL DC medium [RPMI 1640 supplemented with 1% human AB serum (Sigma-Aldrich, Germany) 100 U/mL Penicillin and 100 U/mL Streptomycin, 2 mM L-glutamine and 10 mM HEPES (all Lonza, Switzerland)] in T_175_ cell culture flasks for 1 h at 37°C and 5% CO_2_. Non-adherent cells were collected by washing cell culture flask three times with RPMI 1640 and transferred into a fresh cell culture flask. Cells were allowed to adhere a second time in DC medium. Monocytes in the first and second adherence flasks were cultivated in 30 mL of DC medium supplemented with 800 U/mL GM-CSF (Miltenyi Biotec, Germany) and 250 U/mL IL-4 (Miltenyi Biotec, Germany) for DC differentiation. Three days after adherence, 5 mL fresh DC medium containing 400 U/mL GM-CSF and 250 U/mL IL-4 were added to each T_175_ cell culture flask. The next day, resulting iDCs were activated via adding a cocktail containing GM-CSF (40 U/mL), IL-4 (250 U/mL), IL-1ß (Cell Genix GmbH, Germany; 200 U/mL), IL-6 (Cell Genix GmbH, Germany; 1,000 U/mL), TNF-α (Peprotech, Germany; 10 ng/mL) and PGE_2_ (Pfizer, Germany; 1 μg/mL) to each T_175_ cell culture flask. One point 5 to 2 days after induction of maturation, mDCs were used for subsequent experiments.

### HSV-1 Infection Procedure of mDCs

For HSV-1 infection, a defined cell number of mDCs (2 × 10^6^ mDCs) was mock- or HSV-1-infected in a total volume of 300 μL infection medium (RPMI 1640, 20 mM HEPES) containing a defined amount of HSV-1 virus stock to adjust the respective MOIs as indicated. For UV-irradiation (HSV-1 UV), virus stock was completely inactivated by 8 times applying 0.12 J/cm^2^ in a Vilber Luormat. The infection was performed at 37°C and 350 rpm for 1 h. At 1 h post infection (hpi), cells were centrifuged at 3,390 × g for 2 min and transferred into well-plates containing DC medium (containing 40 U/mL GM-CSF and 250 U/mL IL-4) at a final concentration of 1 × 10^6^ mDCs/mL. Cells were incubated at 37°C and 5% CO_2_ for the indicated time spans. For blocking of phagocytosis by using Cytochalasin D (CytD; Enzo Life Sciences, Germany), mDCs were treated with 2 μM CytD from 1 hpi onwards.

### Coculture Experiments of mDCs and Treatment With a HSV-1 Anti-gB Antibody

For coculture experiments 1 × 10^6^ to 2 × 10^6^ mDCs were mock- or HSV-1 wt-infected (MOI of 5). The infection procedure was performed as described in “HSV-1 infection procedure of mDCs.” At 3 hpi, mock- and HSV-1-infected cells were washed once with PBS and treated with 200 μL of Trypsin-EDTA (Lonza, Switzerland) followed by incubation at 37°C for 1 min. Cells were washed in RPMI and in PBS and transferred into well-plates containing DC medium supplemented with 40 U/mL GM-CSF and 250 U/mL IL-4. Subsequently, at 6 hpi HSV-1-infected cells were cocultured in a 96-round bottom well-plate with mock-treated (each 0.125 × 10^6^ to 0.15 × 10^6^) cells.

The neutralizing HSV-1 anti-gB specific antibody [hu2c (49, 50)] and the anti-CD28 control antibody (BD Pharmingen, purified NA/LE mouse anti-human CD28) were applied at a final concentration of 75 μg/mL. This concentration is based on the efficiency of anti-gB hu2c to neutralize HSV-1 virions used a high MOIs of 50 (data not shown). Cells were harvested 24 hpi and analyzed regarding their IL6R surface expression by flow cytometry as described in “Flow cytometric analyses and fluorescence-activated cell sorting (FACS).”

### Treatment of mDCs With HSV-1-Derived Particles

Mature DCs were incubated with HSV-1-derived particles as follows: 1 × 10^6^ cells were mock- or HSV-1-infected (MOI of 2), incubated with purified H-particles (MOI of 2) or L-particles (viral material corresponding to high MOI). For inactivation of marginal H-particle contaminations, L-particles were inactivated by three times applying 0.12 J/cm^2^ in a Vilber Luormat. The infection was performed as described above. At 1 hpi, mDCs were transferred without centrifugation into DC medium containing 40 U/mL GM-CSF and 250 U/mL IL-4. Cells were harvested 24 hpi and prepared for flow cytometric analyses.

### Flow Cytometric Analyses and Fluorescence-Activated Cell Sorting (FACS)

Cells were harvested at indicated time points post infection by resuspending the cells in the respective 6-, 12- or 24-well-plate. The cells were transferred into 1.5 mL tubes and washed once with staining buffer (PBS containing 2% FCS). The surface staining of IL6R was performed in staining buffer containing an IL6R-specific antibody (Biolegend, PE-Cy7, clone UV4) and LIVE/DEAD Fixable Violet dead cell stain (Life Technologies, CA, USA) for discrimination of living and dead cells at 4°C for 60 min in the dark. Afterwards, cells were washed two times in staining buffer and fixed with 2% PFA in staining buffer. Intracellular IL6R was stained according to manufacturing instructions of the used BD Cytofix/Cytoperm™ kit (BD Biosciences, Germany). As a control, unstained cells were analyzed in parallel. The expression of IL6R was assessed using a FACS Canto II flow cytometer (BD Biosciences, Germany). Data were analyzed with FCS express 5 flow research edition software (*De Novo* Software). The GFP-positive and GFP-negative population was analyzed using different gate sets in the data evaluation software.

For cell sorting based on the GFP signal, cells were harvested 16 hpi and washed once with PBS containing 4% FCS. Afterwards, cells were incubated with DNase for 30 min at 37°C and subsequently stored on ice. Cells were separated into GFP-positive vs. GFP-negative fractions using a BD Aria FACS cell sorter (BD Biosciences, Germany).

### Preparation of Protein Lysates and Immunoblotting

For preparation of protein lysates of sorted cells, pellets were washed once with ice-cold PBS and subsequently resuspended in 35 μL of Natrium-deoxycholat lysis buffer (10% Glycerol, 2 mM EDTA, 137 mM NaCl, 50 mM Tris pH 8.0, 0.5% NP-40) freshly supplemented with 2 mM phenylmethylsulfonyl fluoride, 2 mM sodium orthovanadate, 20 mM sodium fluoride, 0.1 M MgCl_2_ and benzonase and lysed on ice for 20 min. After centrifugation at 13,500 × g at 4°C for 20 min, supernatants were harvested and the protein concentration in each lysate was determined using Bradford protein determination. Subsequently, protein lysates were mixed with 4x Roti-load 1 (final concentration: 1x; Carl Roth GmbH, Germany), followed by denaturation of proteins at 95°C for 10 min. For the preparation of protein lysates of isolated H- and L-particles, the particle solutions were mixed with 4x Roti-Load 1 (final concentration: 1x) and denaturated at 95°C for 10 min immediately after isolation.

Protein lysates derived from cellular or viral material were loaded onto 10% SDS polyacrylamide gels and separated using SDS-PAGE. Afterwards, proteins were transferred onto a nitrocellulose membrane by wet blot transfer. After blocking the membrane in 1x Roti-block (Carl Roth GmbH, Germany) for 1 h at RT, the membrane was incubated with primary antibodies overnight at 4°C. The antibodies were detected via Image Quant and ECL using Amersham ECL Prime Western blotting detection reagent (GE Healthcare, Germany) after the membrane was incubated with the HRP-conjugated secondary antibody. All antibodies are diluted in 1x Roti-block and used as follows: ICP5 antibody (Santa cruz, sc-56989, clone 3B6, 1:1000), gB antibody (Santa cruz, sc-56987, clone 10B7, 1:1000), ICP4 antibody (Santa cruz, sc-56986, clone 10F1, 1:1000), ICP0 antibody (Santa cruz, sc-53070, clone 11060, 1:1000), GAPDH antibody (EMD Millipore Corp., clone MAB374, 1:5000), anti GFP antibody (Santa cruz, sc-9996, clone B-2, 1:1000), polyclonal anti-mouse-IgG HRP-linked (Cell signaling, 1:2500).

### RNA Isolation, cDNA Synthesis and Quantitative Real-Time PCR (qPCR) Analyses

For the isolation of RNA, cells were harvested and washed once with ice-cold PBS. Total RNA was isolated using the QIAshredder kit (Qiagen, Germany) and the RNeasy Plus Mini kit (Qiagen, Germany) according to the manufacturer's instructions. Subsequently, cDNA was transcribed (0.5 μg RNA in a total volume of 20 μL) using Oligo-dT primers and Revert Aid First Strand cDNA Synthesis kit (Invitrogen Thermo Fisher Scientific, Germany). For qPCR analyses, the following mixture was prepared: 5 μL cDNA (concentration of 2.5 ng/μL), 0.8 μL sense primer (10 μM), 0.8 μL of antisense primer (10 μM), 3.4 μL H_2_O and 10 μL of S'Green qPCR 2x Mix (Biozym, Germany).

The following primers were used for qPCR: IL6R sense (5′- TTG TTT GTG AGT GGG GTC CT−3′), IL6R antisense (5′- TGG GAC TCC TGG GAA TAC TG−3′), reference transcripts S14 sense (5′-GGC AGA CCG AGA TGA ATC CTC A-3′), S14 antisense (5′-CAG GTC CAG GGG TCT TGG TCC-3′). All primers were validated according to the Minimum Information for Publication of Quantitative Real-Time PCR Experiments (MIQE) guidelines. First, samples were heated up to 95°C for 3 min. The following 45 cycles were performed as follows: 15 s at 95°C, 15 s at 61°C, and 15 s at 72°C. Afterwards, a melting-curve analysis was performed by subjecting the samples to a temperature ramp (from 65 to 95°C at 0.1°C/s). Quantitative real-time PCR was performed in Touch Thermal Cycler CFX96 real-time system (Bio-Rad, Germany). The final analysis was conducted with CFX Manager 3.0 software (Bio Rad, Germany) and results were normalized to the expression of the S14 reference gene (dCq) and the mock control (ddCq).

### Approvals and Legal Requirements

The permission to perform experiments with human monocyte-derived DCs generated from leukapheresis products of healthy donors was obtained from the local ethics committee (reference number: 184_16Bc). This study was carried out in accordance with the recommendations of the ethics committee of the “Friedrich-Alexander-Universität Erlangen-Nürnberg,” with written informed consent from all subjects. All subjects gave written informed consent in accordance with the Declaration of Helsinki.

### Statistical Analysis

Flow cytometric analyses are displayed as median ± standard deviations (SD) as indicated. For the determination of the significance, data were analyzed using one-way analysis of variance (ANOVA) and Bonferroni's multiple-comparison *post hoc* test or unpaired *t*-test one-trailed as indicated. Significance was accepted for *p* < 0.05. ^****^p ≤ 0.0001; ^***^p ≤ 0.001; ^**^p ≤ 0.01; ^*^p ≤ 0.05; and ns, not significant.

## Results

### Protein and mRNA Expression Levels of IL6R Are Modulated in HSV-1-Infected and Bystander mDCs

**Figure 1:**
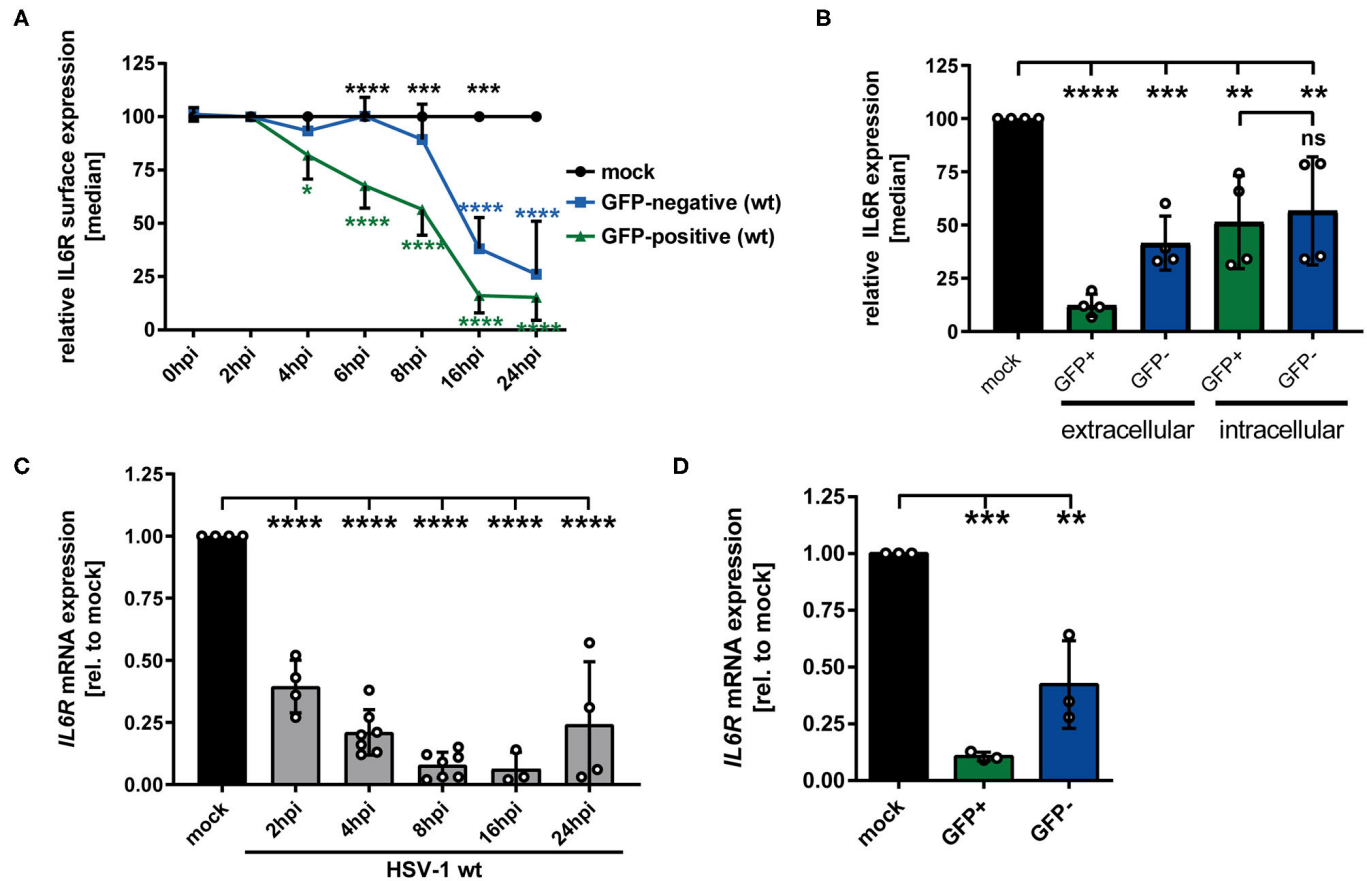
HSV-1 modulation of IL6R expression in/on both GFP-positive directly-infected and GFP-negative bystander mDCs. **(A)** Mature DCs were mock- or HSV-1 wt-infected (MOI of 0.65) and harvested at indicated time points post infection (0–24 hpi). IL6R surface expression was analyzed on mock (set to 100%, black line), GFP-positive (green line) and GFP-negative (blue line) mDCs by flow cytometry. The experiment was performed at least four times with cells derived from different healthy donors. Green asterisks indicate statistical analyses of GFP-positive vs. mock treated cells, blue asterisks the statistical analyses of GFP-negative vs. mock treated cells and black asterisks the statistics of GFP-negative vs. GFP-positive cells. **(B)** Mature DCs were mock- or HSV-1 wt-infected (MOI of 0.6) and harvested at 24 hpi. Cells were stained using an IL6R-specific antibody to assess extracellular and intracellular expression levels. IL6R surface expression was analyzed on mock (set to 100%, black line), GFP-positive (green line) and GFP-negative (blue line) mDCs by flow cytometry. Statistical analyses between GFP-positive and GFP-negative samples regarding intracellular IL6R expression levels were carried out applying the unpaired *t*-test. **(A,B)** Distinction between infected and uninfected mDCs is based on the GFP signal and the used gates in data evaluation software FCS Express 5, which are specific for either the GFP-positive or GFP-negative population. **(C)** Mature DCs were mock or HSV-1 wt-infected (MOI of 2, unsorted cells) and harvested at indicated time points post infection (2–24 hpi). RNA was isolated and qPCR was performed. Relative IL6R mRNA expression levels are normalized to S14 and are shown relative to the respective mock condition. The experiment was performed four times with cells isolated from different healthy donors. **(D)** Mature DCs were mock- or HSV-1 wt-infected (MOI of 0.6) and harvested 16 hpi. Subsequently, cells were sorted based on their GFP expression into GFP-positive and GFP-negative mDCs. RNA was isolated, transcribed into cDNA and used for subsequent qPCR experiments. Relative IL6R mRNA expression levels are normalized to S14. IL6R transcript levels are shown relative to the respective mock condition. The experiment was performed three times with cells derived from different healthy donors. Error bars indicate SD. Significant changes to mock were analyzed using a one-way ANOVA and Bonferroni multiple comparison *post hoc* tests and are indicated by asterisks (*p ≤ 0.05, **p ≤ 0.01, ***p ≤ 0.001, ****p ≤ 0.0001). Not significant changes (*p* > 0.05) are depicted as “ns”.

Previous studies showed that HSV-1 specifically modulates different proteins expressed by mDCs, e.g., CD83, CCR7, CXCR4, and CYTIP, to hijack important mDC functions (18, 21, 22). Since IL-6 signaling in mDCs is a critical pathway during the immune response, we investigated whether and how HSV-1 modulates IL6R expression by mDCs. Thus, in a first approach, mDCs were HSV-1 infected using an EGFP expressing reporter HSV-1 strain (herein designated as HSV-1 wt) at a low MOI of 0.65, to subsequently distinguish between HSV-1-infected GFP-positive cells and uninfected GFP-negative bystander mDCs. Using a data evaluation software, we can specifically gate on the GFP-negative and GFP-positive fraction and individually examine both populations. Mock-infected mDCs served as controls. Directly-infected and uninfected bystander mDCs were monitored regarding their IL-6 receptor (IL6R) surface expression during HSV-1 infection kinetics via flow cytometry. As early as 6–8 hpi, clearly diminished IL6R surface expression levels were detected on HSV-1-infected GFP-positive mDCs compared to mock cells (Figure 1A, green line). This effect increased dramatically within time post infection, resulting in an almost complete loss of IL6R expression on the cell surface of directly HSV-1-infected mDCs at 24 hpi compared to the mock control. Surprisingly, reduced IL6R surface expression levels, compared to mock-infected mDCs, were not only observed on directly-infected, but also on uninfected GFP-negative bystander mDCs. Noteworthy, on uninfected bystander mDCs this effect occurred in a timely-delayed fashion as well as less pronounced compared to directly-infected GFP-positive mDCs (Figure 1A, blue line). Since IL6R was described to be predominantly present in intracellular compartments, e.g., recycling endosomes, we were interested whether HSV-1 affects intracellular IL6R expression levels (29). Using the same experimental setup as described above, flow cytometric analyses revealed decrease levels of intracellular IL6R in directly GFP-positive and uninfected bystander mDCs compared to mock-infected mDCs (Figure 1B). In contrast to the analyses of the IL6R surface expression, intracellular expression levels were similarly regulated in GFP-positive and GFP-negative mDCs (Figure 1B, intracellular).

To further elucidate whether HSV-1-mediated modulation of IL6R surface expression is also present on mRNA levels, qPCR analyses were performed using cDNA derived from mock- or HSV-1 wt-infected mDCs (2-24 hpi). Consistent with the IL6R protein modulation on the cell surface, also IL6R specific transcripts significantly dropped in HSV-1-infected mDCs (Figure 1C). In contrast to IL6R modulation on protein levels, already at 2 hpi IL6R mRNA decreased to ~50%, when compared to mock treated cells.

Moreover, IL6R mRNA expression levels were separately analyzed in directly-infected GFP-positive and GFP-negative bystander mDCs at 16 hpi. For this, mDCs were infected with HSV-1 wt (MOI of 0.6) followed by FACS based on the GFP signal as an indicator of direct infection. As shown in Figure 1D, IL6R transcription levels were not only decreased in GFP-positive but also in GFP-negative bystander mDCs, however to a lesser degree. Taken together, these data revealed that IL6R surface expression as well as mRNA expression is significantly hampered during an HSV-1 infection, both on/in directly-infected and uninfected bystander mDCs.

### Modulation of IL6R Surface Expression Is Transmittable From Directly HSV-1-Infected to Uninfected Bystander mDCs

**Figure 2:**
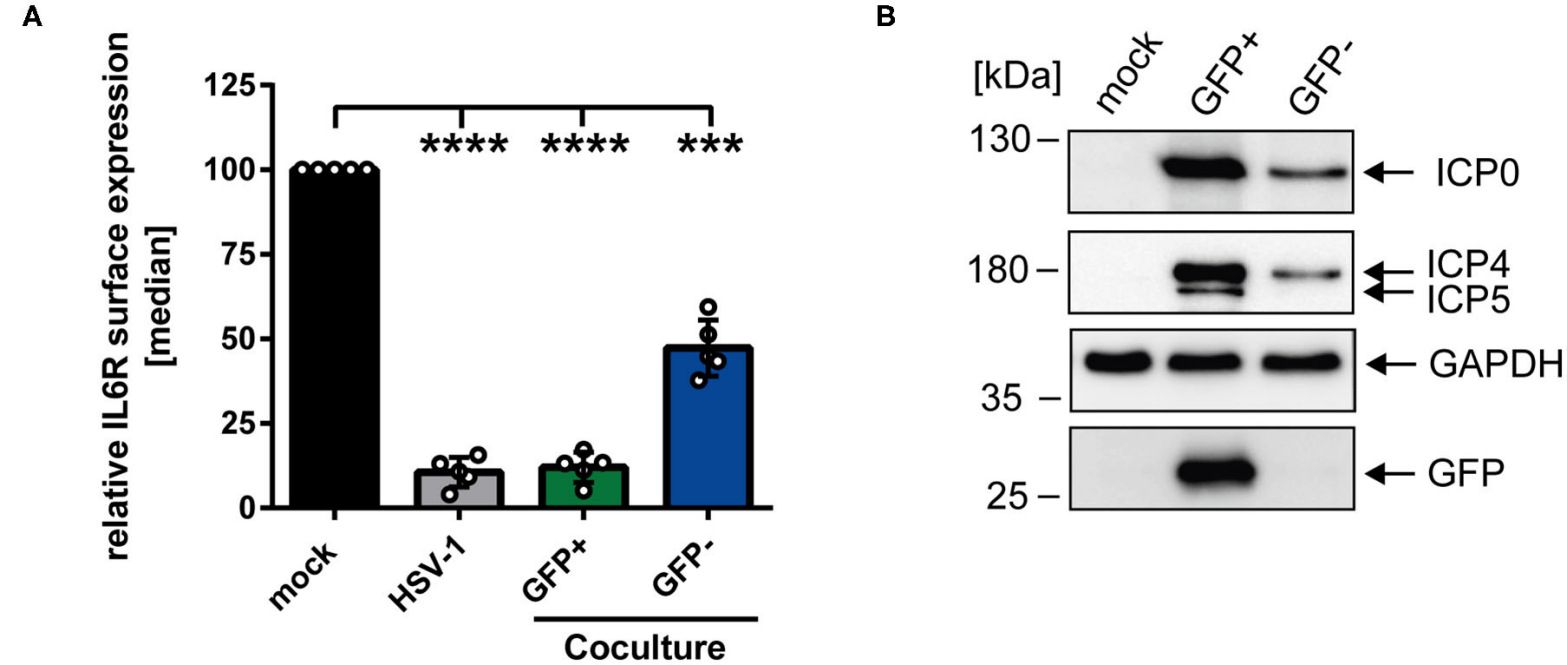
HSV-1-mediated IL6R modulation is transmittable from directly-infected GFP-positive to uninfected GFP-negative bystander mDCs. **(A)** Mature DCs were mock- or HSV-1 wt-infected (MOI of 5), all samples were trypsinized at 3 hpi and subsequently cocultured with uninfected mDCs at 6 hpi. HSV-1 wt-infected mDCs were included as a positive control (“HSV-1,” gray bar). Cells were harvested 24 hpi, stained with an IL6R-specific antibody and analyzed by cytometry. Distinction between infected and uninfected mDCs is based on the GFP signal and the used gates in data evaluation software FCS Express 5, which are specific for either the GFP-positive or GFP-negative population. IL6R surface expression is shown as median and normalized to the mock condition. The experiment was performed five times with cells derived from different healthy donors. Error bars indicate SD. Significant changes were analyzed using a one-way ANOVA and Bonferroni multiple comparison *post hoc* tests and are indicated by asterisks (***p ≤ 0.001, ****p ≤ 0.0001). **(B)** Mature DCs were mock- or HSV-1 wt-infected (MOI of 0.6) and harvested 16 hpi. Subsequently, cells were sorted based on their GFP expression into GFP-positive and GFP-negative mDC fractions. Protein lysates of sorted cells were analyzed using Western blot for detection of ICP0, ICP4, ICP5, GFP, and GAPDH as loading control. The experiment was performed three times with cells derived from different healthy donors.

To gain further insights regarding the reduced IL6R surface expression levels on bystander cells compared to mock-infected mDCs, coculture experiments were performed. HSV-1-infected mDCs were harvested 6 hpi, incubated with trypsin to remove surface-bound virions, and subsequently cocultured with uninfected mDCs. At 24 hpi, cells were analyzed using an IL6R-specific antibody and examined via flow cytometry. Figure 2A illustrates that HSV-1 did not only modulate IL6R surface expression on directly-infected GFP-positive mDCs but also on uninfected GFP-negative bystander mDCs compared to the mock control. Based on these results, we conclude that IL6R surface expression was also hampered on cocultured mDCs, which were not directly exposed to infectious virus prior to cocultivation. In addition, we investigated the effect of trypsin on the IL6R surface expression, and found no influence on its expression (data not shown). Next, we analyzed whether viral proteins can be transferred from HSV-1-infected mDCs to bystander mDCs. Therefore, mDCs were infected with HSV-1 wt (MOI of 0.6) and sorted in respect to their GFP expression into GFP-positive and GFP-negative fractions 16 hpi. Western blot analyses of these sorted cells revealed that except from the capsid-associated infected cell protein (ICP) 5, viral proteins, such as ICP0 and ICP4, were transferred to bystander mDCs (Figure 2B). Taken together, these results indicate that viral components are transmitted from directly HSV-1-infected mDCs to uninfected bystander mDCs triggering IL6R modulation.

**Figure 3:**
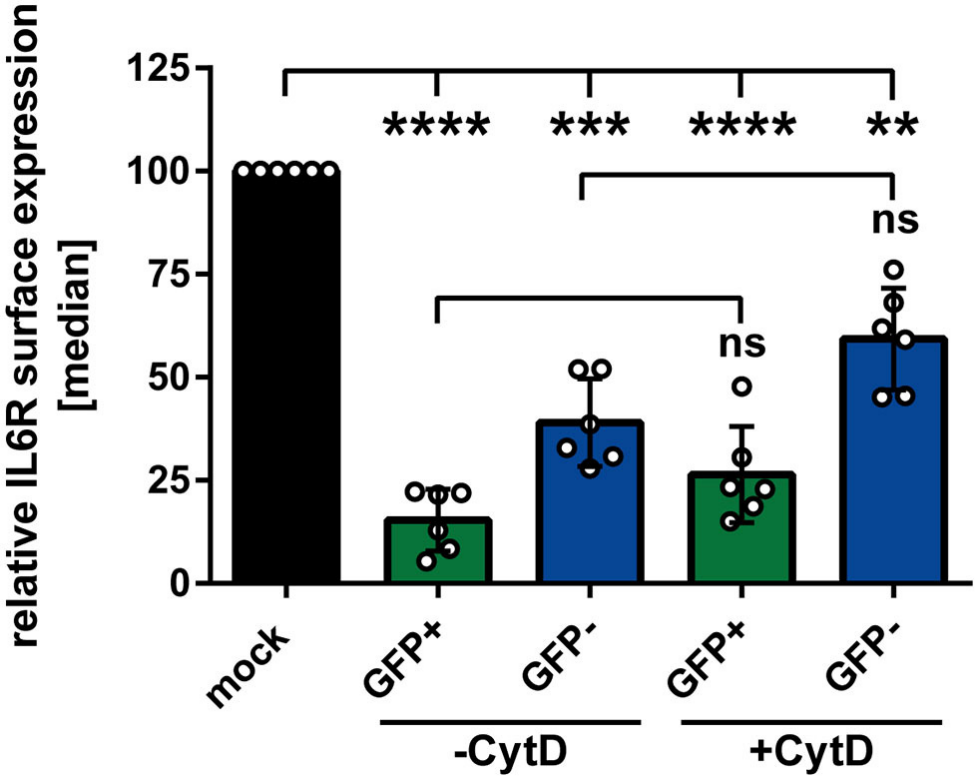
Modulation of IL6R surface expression on bystander mDCs is independent from phagocytosis. Mature DCs were mock- or HSV-1 wt-infected (MOI of 0.65), treated with or without Cytochalasin D (CytD) from 1 hpi ongoing and harvested 16 hpi. Cells were stained with an IL6R-specific antibody for subsequent flow cytometric analyses. Distinction between infected and uninfected mDCs is based on the GFP signal and the used gates in data evaluation software FCS Express 5, which are specific for either the GFP-positive or GFP-negative population. The median of IL6R surface expression was normalized to mock expression levels. The experiment was performed six times with cells derived from different healthy donors. Error bars indicate SD. Significant changes were analyzed using a one-way ANOVA and Bonferroni multiple comparison *post hoc* tests and are indicated by asterisks (**p ≤ 0.01, ***p ≤ 0.001, ****p ≤ 0.0001). Not significant changes (*p* > 0.05) are depicted as “ns”.

In the next experiment we aimed to analyze whether or not phagocytosis plays a role during the observed HSV-1-mediated IL6R modulation on uninfected bystander mDCs. Apoptosis of HSV-1 infected mDCs might lead to their engulfment by bystander mDCs. This could trigger the reduction of IL6R surface expression on these uninfected bystander mDCs, compared to mock-infected mDCs. To proof or disproof this hypothesis, mDCs were mock- or HSV-1 wt-infected, treated with the phagocytosis inhibitor Cytochalasin D (CytD), or DMSO as a control, and harvested 16 hpi. In advanced, the successful inhibition of the phagocytosis by CytD was verified (data not shown). As shown in Figure 3, HSV-1 induced a significant modulation of IL6R surface expression on directly-infected as well as on uninfected bystander mDCs, also in the presence of the phagocytosis inhibitor CytD. Hence, our results demonstrate that HSV-1 induces a phagocytosis-independent modulation of IL6R expression on bystander mDCs.

### HSV-1 Modulates IL6R Expression Levels Also via a Replication-Independent Mechanism

**Figure 4:**
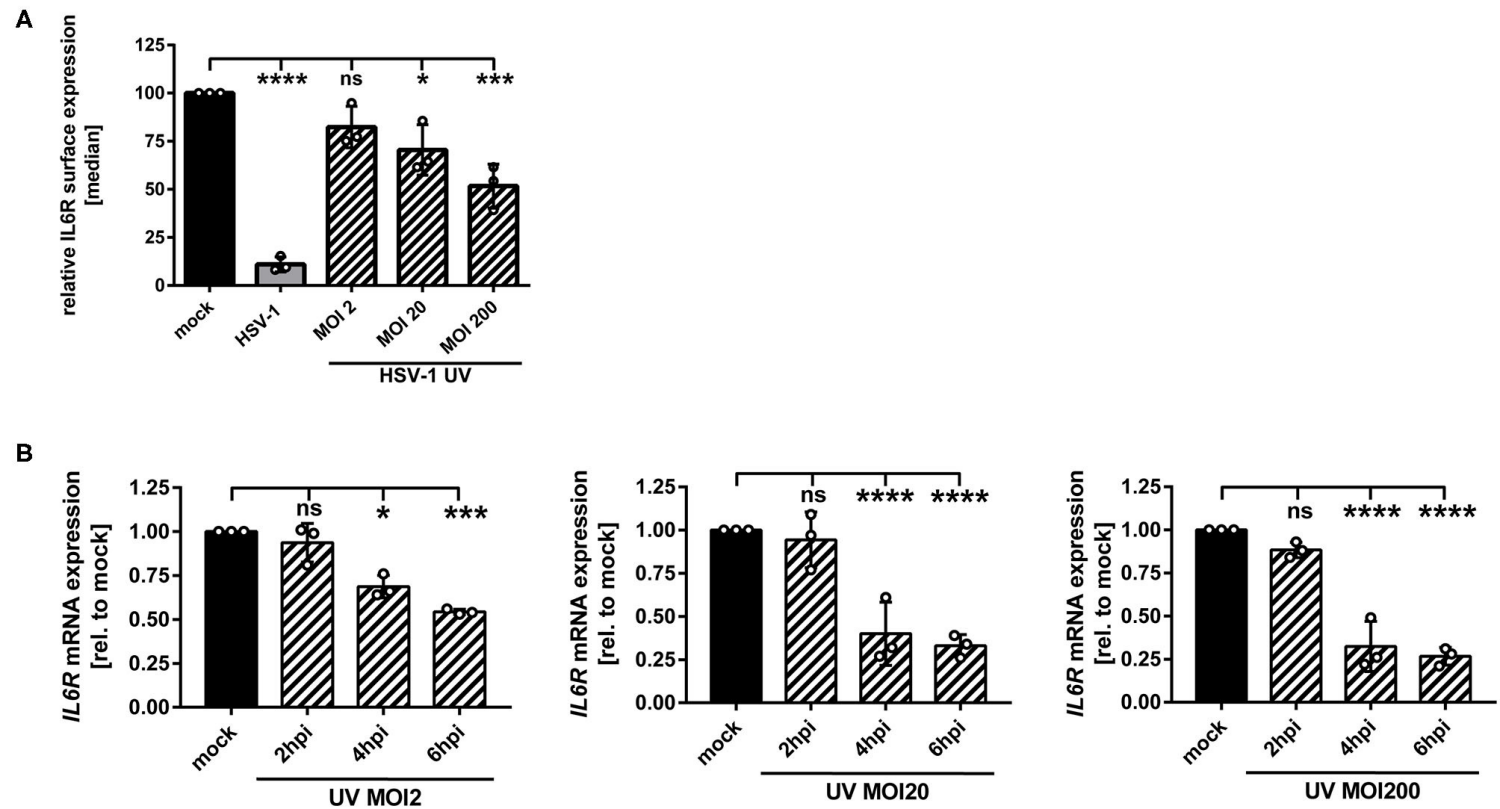
HSV-1 induces IL6R modulation via a replication-independent mechanism. **(A,B)** Mature DCs were mock-treated (black bars) or HSV-1 wt-infected (MOI of 2, gray bars) or incubated with UV-inactivated HSV-1 virions using the indicated MOIs (viral material corresponding to MOI of 2, 20, 200, irradiated 8 times applying 0.12 J/cm^2^, black dashed bars). **(A)** Cells were harvested 24 hpi, stained with an IL6R-specific antibody and surface expression was analyzed via flow cytometry. The median of IL6R surface expression was normalized to the expression levels of mock treated cells. The experiment was performed three times with cells derived from different healthy donors. **(B)** Cells were harvested 2, 4, 6 hpi. RNA was isolated and qPCR was performed using transcribed cDNA. Relative IL6R mRNA expression levels are normalized to S14 and shown relative to the respective mock condition. The experiment was performed three to seven times with cells derived from different healthy donors. Error bars indicate SD. Significant changes to mock were analyzed using a one-way ANOVA and Bonferroni multiple comparison *post hoc* tests and are indicated by asterisks (*p ≤ 0.05, ***p ≤ 0.001, ****p ≤ 0.0001). Not significant changes (*p* > 0.05) are depicted as “ns”.

We have described so far that replication-competent HSV-1 virions induce the IL6R modulation on/in mDCs (Figure 1). In order to assess whether this can also occur in a replication-independent manner, mDCs were inoculated with UV-inactivated HSV-1 wt virions (8 × 0.12 J/cm^2^), using increasing MOIs ranging from 2 to 200. Given the fact that UV-irradiated HSV-1 virions are replication-incompetent, there is a lack of *de novo* viral protein synthesis and only viral proteins already present in the tegument during inoculation of mDCs are capable of modulating the expression of cellular proteins. As controls, mDCs were mock treated or infected with intact HSV-1 wt virions (MOI of 2) and for flow cytometric analyses, cells were harvested 24 hpi (Figure 4A). When using moderate MOIs (e.g., viral material corresponding to an MOI of 2), inoculation of mDCs with UV-inactivated HSV-1 only marginally affected IL6R surface expression relative to mock-infected mDCs. However, higher amounts of UV-inactivated HSV-1 (viral material corresponding to an MOI of 20 or 200) significantly impaired IL6R surface expression. In particular, inoculation of mDCs with UV-inactivated viral material corresponding to an MOI of 200 caused an ~50% reduction of IL6R surface expression, relative to mock-treated controls.

In addition, we performed IL6R mRNA expression analyses at early time points post infection. As shown in Figure 4B, also in mDCs treated with HSV-1 wt UV-irradiated virions (viral material corresponding to an MOI of 2) significantly lower levels of IL6R mRNA were detected at 4–6 hpi. This effect increased by using higher amounts of UV-irradiated virions. Therefore, we conclude that in mDCs HSV-1 regulates IL6R expression levels also via a replication-independent mechanism that might be triggered by at least one virus-encoded protein, which is incorporated into HSV-1 virions.

### HSV-1-Derived L-Particles Are Sufficient to Modulate IL6R Surface Expression on Bystander mDCs

**Figure 5:**
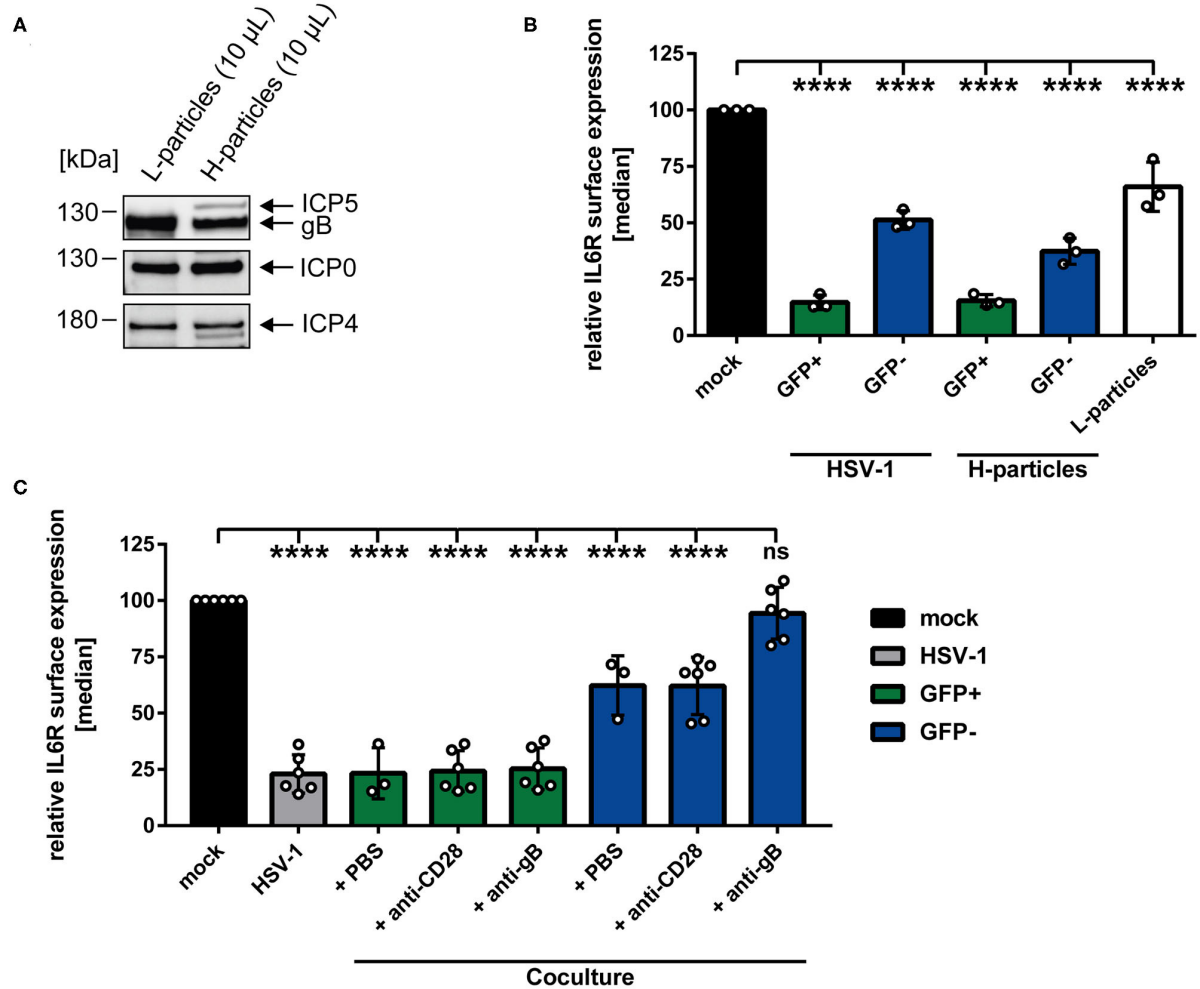
HSV-1-mediated IL6R modulation is transmittable via L-particles from directly-infected GFP-positive to uninfected GFP-negative bystander mDCs. **(A)** Western blot analyses of purified L- and H-particles derived from BHK21 cells. Antibodies specific for ICP0, ICP4, ICP5 and gB were used. One exemplary experiment out of ten is shown. **(B)** Mature DCs were either infected with HSV-1 wt (MOI of 0.65), purified H-particles (MOI of 0.65) or treated with L-particles (viral material corresponding to high MOI, irradiated three times applying 0.12 J/cm^2^, white bar). Cells were harvested 24 hpi and analyzed for their IL6R surface expression via flow cytometry. The experiment was performed three times with cells derived from different healthy donors. **(C)** Mature DCs were mock- or HSV-1 wt-infected (MOI of 5) and all samples were trypsinized at 3 hpi. Subsequently, infected cells were cocultured with uninfected mDCs in the presence of an anti-gB specific antibody, an anti-CD28 control antibody or PBS (ctrl) at 6 hpi. HSV-1 wt-infected mDCs, cultured for 24 h, were included as a positive control (“HSV-1,” gray bar). Cells were harvested 24 hpi and stained with an IL6R-specific antibody and analyzed by flow cytometry. GFP-positive directly-infected mDCs (green bars) and GFP-negative uninfected bystander mDCs (blue bars) in the coculture are depicted for each condition. The experiment was performed three to six times with cells from different donors. **(B,C)** Distinction between infected and uninfected mDCs is based on the GFP signal and the used gates in data evaluation software FCS Express 5, which are specific for either the GFP-positive or GFP-negative population. Error bars indicate SD. Significant changes to mock were analyzed using a one-way ANOVA and Bonferroni multiple comparison *post hoc* tests and are indicated by asterisks (****p ≤ 0.0001).

Having demonstrated that IL6R regulation not only occurs on directly-infected GFP-positive, but also on GFP-negative bystander mDCs in coculture experiments, we hypothesized that a soluble factor transmitted from HSV-1-infected to uninfected bystander mDCs may be responsible for IL6R modulation on bystander mDCs. It was reported recently that the production of infectious H-particles is hampered in HSV-1-infected mDCs, thereby predominantly releasing non-infectious L-particles (13). Furthermore, L-particles are described to be sufficient to downmodulate CD83 protein levels on bystander cells (16). Thus, it was tempting to speculate that L-particles might also play a major role in the HSV-1-mediated IL6R regulation on mDCs. To verify this, non-infectious L-particles and mature virions (H-particles) were separately isolated from HSV-1 wt-infected BHK21 cells. Subsequently, both particle preparations were characterized regarding their presence or absence of specific viral proteins, such as ICP5, the major capsid protein, present in infectious H-particles and as expected absent in L-particles (Figure 5A). Additional viral proteins, such as ICP0, ICP4 and glycoprotein B (gB) are present in both particle types.

Regarding the observed IL6R surface regulation on the GFP-negative bystander mDCs and based on our time kinetic analyses, the involvement of L-particles, which are present in the HSV-1 stock preparations, cannot be excluded (Figure 1, blue line). Since L-particles are devoid of the viral capsid and thus the genome, a distinction between mDCs affected by L-particle of a given virus stock and GFP-negative bystander mDCs is not possible. However, to assess the involvement of L-particles, contained in the virus stock, mDCs were either HSV-1 wt-infected or exposed to purified H-particles (MOI of 2) for 24 h (Figure 5B). The results of this experiment, depicted in Figure 5B, revealed that IL6R surface expression on GFP-negative bystander mDCs was equally affected by the infection with purified H-particles as with the used HSV-1-virus stock, containing both H- and L-particles (“H-particles” blue bar). Thus, we conclude that L-particles contained in the virus stock do not evidently affect IL6R surface expression on uninfected GFP-negative mDCs. Finally and to proof that L-particles are able to diminish IL6R surface expression on uninfected bystander mDCs, mDCs were inoculated with purified L-particles, and IL6R surface expression was analyzed as shown in Figure 5B (white bar). Importantly, treatment of mDCs with purified L-particles was able and sufficient to significantly reduce IL6R expression on mDCs, compared to mock-infected mDCs.

Having shown that L-particles are indeed involved in IL6R regulation, we investigated if a neutralizing anti-gB specific antibody is able to interfere with this L-particle-mediated effect on uninfected bystander mDCs. The HSV-1 encoded surface molecule gB is essential for the attachment of HSV-1 to the host cell via binding to heparan sulfate proteoglycans (HSPG) and paired Ig-like type 2 receptor alpha (PILRα) (7, 51). Both HSPG and PILRα are expressed on DCs (52, 53). Thus, a humanized monoclonal anti-gB antibody, previously identified as potent inhibitor of free HSV-1 virions and HSV-1 cell-to-cell spread, was applied in a coculture experiment (49). Our hypothesis was that this antibody blocks the transfer and uptake of L-particles from directly-infected to uninfected bystander mDCs, and thereby inhibits L-particle-mediated modulation of IL6R on bystander mDCs. Interestingly, diminished levels of IL6R surface expression on the cell surface of GFP-negative bystander mDCs was significantly restored by this anti-gB antibody relative to mock-treated mDCs (Figure 5C, blue bar, + anti-gB). An anti-CD28 antibody or PBS (ctrl) were used as negative controls and did not restore IL6R surface expression on GFP-negative bystander mDCs (Figure 5C, blue bars + PBS and + anti-CD28). Likewise, the IL6R expression on directly HSV-1-infected mDCs was not affected by the anti-gB antibody or controls (Figure 5C, green and gray bars). In summary, these results clearly support the conclusion that L-particles generated by mDCs during HSV-1 infection, transmit viral proteins to uninfected bystander mDCs, thereby modulating IL6R surface expression.

### The HSV-1 Encoded Vhs Protein Is Involved in IL6R Reduction in Directly-Infected and Uninfected Bystander mDCs

**Figure 6:**
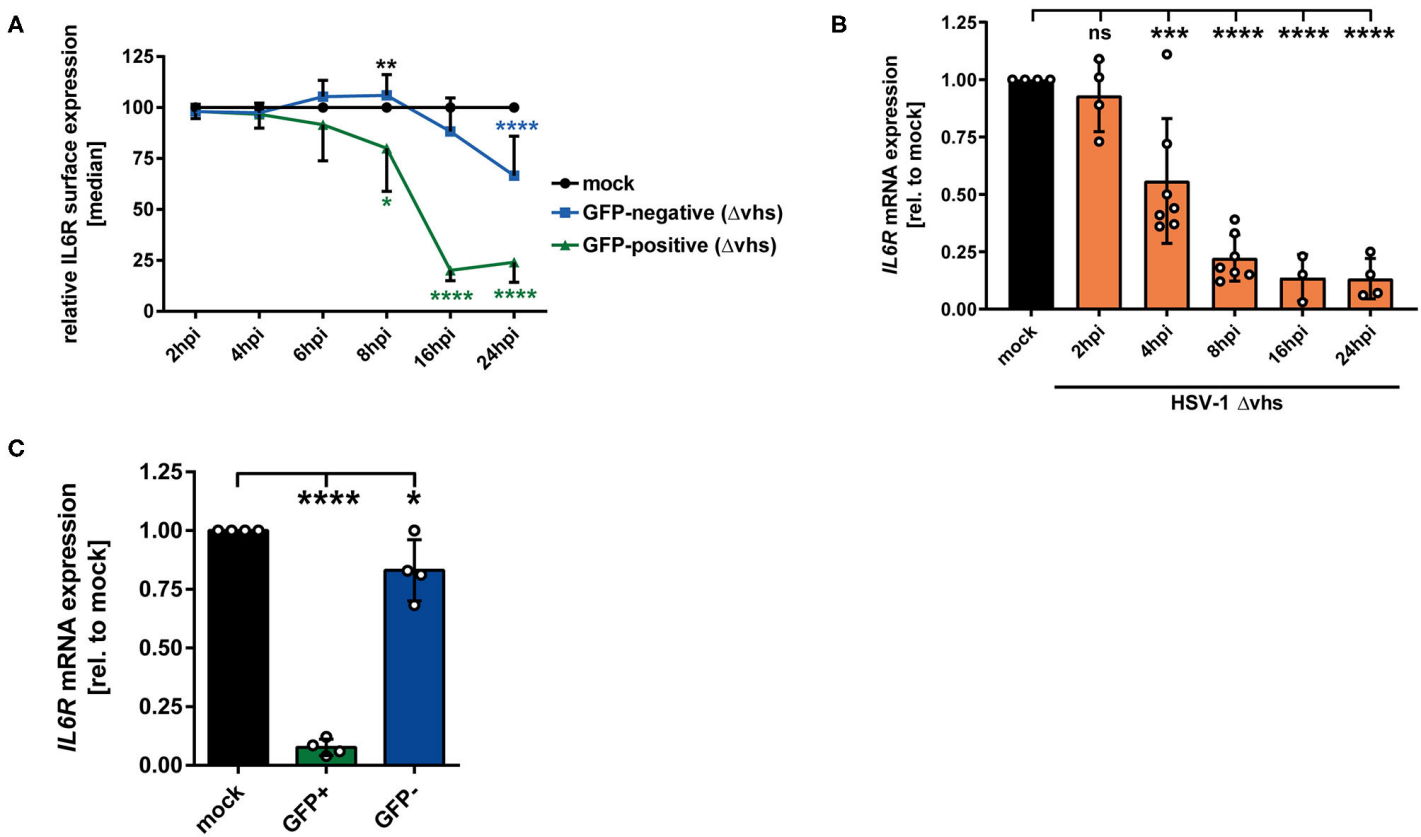
HSV-1 viral protein vhs is partially involved in IL6R regulation in infected mDCs as well as in uninfected bystander mDCs. **(A)** Mature DCs were mock- or HSV-1 Δvhs-infected (MOI of 0.6) and harvested at indicated time points post infection. IL6R surface expression was analyzed on mock (set to 100%, black line), GFP-positive (green line) and GFP-negative (blue line) HSV-1 Δvhs-infected mDCs by flow cytometry. Distinction between infected and uninfected mDCs is based on the GFP signal and the used gates in data evaluation software FCS Express 5, which are specific for either the GFP-positive or GFP-negative population. This experiment was performed three times with cells derived from different healthy donors. Green asterisks indicate statistical analyses of GFP-positive to mock-treated mDCs, blue asterisks the statistical analyses of GFP-negative to mock-treated mDCs and, black asterisks the statistics of GFP-negative to GFP-positive conditions. **(B)** Mature DCs were mock treated (black bar), or HSV-1 Δvhs-infected (MOI of 2, orange bars) and harvested at indicated time points post infection (2–24 hpi). RNA was isolated and qPCR was performed using transcribed cDNA. Relative IL6R mRNA expression levels are normalized to S14 and are shown relative to the respective mock condition. This experiment was performed three to seven times with cells derived from different healthy donors. **(C)** Mature DCs were mock- or HSV-1 Δvhs-infected (MOI of 0.6) and harvested 16 hpi. Subsequently, cells were sorted based on their GFP expression into GFP-positive and GFP-negative mDCs fraction. RNA was isolated and transcribed cDNA used for subsequent qPCR experiments. Relative IL6R mRNA expression is normalized to S14 and transcript levels are shown relative to the respective mock condition. Data points are based on the analyses of cells from four different healthy donors. Error bars indicate SD. Significant changes were analyzed using a one-way ANOVA and Bonferroni multiple comparison *post hoc* tests and are indicated by asterisks (*p ≤ 0.05, **p ≤ 0.01, ***p ≤ 0.001, ****p ≤ 0.0001). Not significant changes (*p* > 0.05) are depicted as “ns”.

In order to elucidate which viral protein contributes to IL6R regulation during HSV-1 infection, different HSV-1 strains lacking specific viral proteins were tested regarding their impact on IL6R modulation during mDC infection. Since all tested HSV-1 deletion strains (ΔICP0, ΔICP27, ΔICP34.5/ΔICP47), except HSV-1 Δvhs, affected IL6R surface expression on directly-infected mDCs comparable to HSV-1 wt (data not shown), we focused on the HSV-1 virus stock ablated for virion host shutoff (vhs) protein expression. The vhs gene encodes for a viral endoribonuclease which is important for the degradation of both cellular and viral mRNAs (44, 45). Concerning the involvement of vhs, mDCs were mock- or HSV-1 Δvhs-infected (MOI of 0.6) and harvested at different time points 2–24 hpi. The HSV-1 Δvhs-infected mDCs also expresses EGFP, which allows the distinction between GFP-positive infected and GFP-negative bystander mDCs when using low MOI of 0.6. On GFP-positive HSV-1 Δvhs-infected mDCs IL6R surface expression was strongly impaired, becoming significant at 8 hpi (Figure 6A, green line). In comparison, in HSV-1 wt-infected mDCs, this effect was already observable at 4 hpi (see Figure 1A, green line). Nevertheless, at later time points (16–24 hpi) IL6R surface expression on HSV-1 Δvhs-infected mDCs was reduced to levels comparable to HSV-1 wt. In contrast, the IL6R expression levels on uninfected GFP-negative bystander mDCs were notably different among an HSV-1 wt and HSV-1 Δvhs infection (Figures 1A, 6A; blue lines). While GFP-negative bystander mDCs displayed 60–70% lower IL6R surface expression in the wt infection 16–24 hpi, Δvhs-infected mDCs express ~25% reduced IL6R levels, relative to the respective mock control.

The modulation of IL6R expression was also analyzed on mRNA level. As depicted in Figure 6B, IL6R was impaired in a time-dependent mechanism in HSV-1 Δvhs-infected mDCs becoming statistically significant at 4 hpi. In contrast, HSV-1 wt-infected mDCs already exhibited a severe reduction of IL6R mRNA levels after 2 h of infection (Figure 1B). In addition, IL6R transcript levels were also analyzed in sorted GFP-positive infected and GFP-negative bystander mDCs (Figure 6C). These data clearly demonstrate that vhs interferes with IL6R mRNA levels in bystander mDCs. In conclusion, our observations indicate that the viral tegument protein vhs plays an important role in the IL6R regulation in/on directly-infected mDCs (early time points) and even more important in the regulation in/on uninfected bystander mDCs.

## Discussion

HSV-1 constitutes a very successful human pathogenic virus, being well-equipped for surviving in and modulating its host cells. Concerning this, HSV-1 has acquired several immune evasion mechanisms in different cell types, e.g., impairment of MHC class I and II presentation (54–56), downregulation of CD83 on mDCs (16, 18) or inhibition of mDC migration (22). In the present study, we report that HSV-1 also targets the IL-6 signaling pathway in mDCs by interfering with IL6R expression. The IL-6 signaling pathway involves the binding of the pleiotropic cytokine IL-6 to its cognate IL-6 receptor complex, composed of gp130 and the membrane-bound IL-6 receptor (IL6R) component, and plays a crucial role during migration, pro-inflammatory signaling and apoptosis (24, 25, 34).

The data presented in this study demonstrate that IL6R expression levels were significantly reduced on HSV-1-infected mDCs, compared to mock controls, as early as 4 hpi, with an almost complete loss of IL6R on the cell surface at 24 hpi (Figure 1A, green line). The modulation of the IL6R expression was also true for intracellular protein levels (Figure 1B). Noteworthy, flow cytometric analyses further revealed significantly impaired IL6R surface expression on GFP-negative bystander mDCs (Figure 1A, blue line). In comparison to directly HSV-1-infected mDCs, IL6R surface expression on bystander mDCs was decreased timely-delayed and less pronounced, relative to mock-infected mDCs. In addition, IL6R mRNA expression levels were reduced as early as 2 hpi, indicating an HSV-1-mediated transcriptional regulation with very fast kinetics (Figure 1C). The regulation of mRNA expression was also significant in GFP-negative bystander mDCs 16 hpi, however, and in agreement with the protein levels (Figure 1A), less pronounced as in GFP-positive cells (Figure 1D).

Coculture experiments performed in the present study suggested that directly HSV-1-infected mDCs release *de novo* produced factors into the supernatant which induce the IL6R modulation on bystander mDCs (Figure 2A). Noteworthy in this respect, virus-derived components in the supernatants are not phagocytized by uninfected bystander mDCs (Figure 3). Our finding that inoculation of mDCs with UV-inactivated virions also hampered IL6R surface expression provides evidence that viral replication is not absolutely essential for the observed reduced IL6R surface expression levels mediated by HSV-1. This suggests that viral proteins incorporated into virions are sufficient to partially mediate the observed effect (Figure 4).

A recent publication shows that, due to the intrinsic inhibition of autophagic turnover and thus lamin degradation, HSV-1 capsids are trapped within the nucleus of mDCs and thus HSV-1-infected mDCs predominantly release non-infectious L-particles (13). These L-particles were described to downregulate functionally important surface molecules, such as CD83, on uninfected bystander mDCs (16). In general, L-particles, with the exception of the capsid and thus the viral genome, are similarly assembled as mature virions (57, 58). Regarding this, *de novo* L-particle synthesis by directly HSV-1-infected mDCs might explain the time-delayed onset of IL6R modulation on the cell surface of uninfected bystander mDCs (Figure 1A, blue line). Our finding that IL6R expression is also affected on GFP-negative bystander mDCs, in the context of an infection using pure H-particles (MOI of 0.65), supports the hypothesis that L-particles are responsible for this IL6R regulation. Since the treatment of mDCs with pure H-particles (mature virions) reduced IL6R surface expression on bystander mDCs, compared to mock-infected mDCs, we excluded the possibility that the observed IL6R effects on bystander mDCs are due to L-particles contained in the HSV-1 stock preparations (Figure 5B, “H-particles”). Recently, Russel *et al*. have shown that L-particles derived from HSV-1 strain Sc16-infected HaCaT or BoHV-1 strain P8-2-infected MDBK cells contain a plethora of viral tegument proteins and the vast majority of viral glycoproteins (59). Given the overall homology among various HSV-1 strains (60), it is very likely that L-particles derived from HSV-1 strain HSV-1 syn 17+/CMV-EGFP/UL43-infected mDCs, are similar to the composition of particles reported by others (59, 61, 62). We recently analyzed the composition of L-particles derived from HSV-1-infected mDCs as well as from BHK21 cells by mass spectrometry, and found a wide range of viral proteins incorporated into L-particles derived from either of both cell types (63). Despite of the blocked H-particle production by HSV-1-infected mDCs, we hypothesize that L-particles are produced and released to transfer a variety of HSV-1-encoded proteins to the cellular microenvironment and to shape bystander cells in benefit of the virus. Another particle type generated during an HSV-1 infection are so called defective interfering particles (DIPs), which spontaneously arise and are thus contained in virus stocks of multiple passages (64). Due to their mutations in the viral genome, DIPs are replication incompetent on their own, but still contain viral DNA (65). Based on the previous publication, viral capsid and thus the viral genome is trapped inside the nucleus of HSV-1 infected mDCs. Consequently, only L-particles, which are devoid of the genome, are released by mDCs (13). Therefore, it is more likely that L-particles elicit the observed IL6R modulation and not DNA containing DIPs.

However, only very little was known regarding L-particles generated by mDCs, including how they are transferred to and modulate bystander cells during infection. Here we provide for the first time experimental evidence, that L-particles are responsible for the IL6R modulation on uninfected bystander mDCs. To further proof that transferred L-particles are responsible for this effect, we inhibited the transfer of L-particles to bystander mDCs using an anti-gB specific antibody (49, 50). In general, HSV penetrates its host cells via different entry routes, whereby the main entry routes are the pH independent fusion with the plasma membrane of the host cell (66), or endocytosis-mediated entry (67). For virus entry and attachment to the host cell, the surface molecule gB is essential (7, 51). In addition, mDCs express specific receptors, such as HVEM (68), PILRα (51) and DC-SIGN (53), to which HSV-1 specific glycoproteins, e.g., gB and gD, bind and thereby induce penetration of the virus into the cell (69). Thus, a gB specific antibody was applied to inhibit the transfer and attachment of HSV-1-derived particles. Supporting our hypothesis, this antibody interfered with the transmission of released L-particles from infected to uninfected bystander mDCs during our coculture infection experiments and inhibited IL6R modulation on these cells (Figure 5C). Notwithstanding, the observed HSV-1-induced effects on IL6R surface expression are mainly regulated by H-particles and to a lesser extent by L-particles.

To investigate whether the lower IL6R surface expression levels might be due to specific mRNA regulation upon an HSV-1 infection of mDCs, we performed qPCR analyses of IL6R transcripts. Since IL6R mRNA expression levels were significantly altered by HSV-1 (Figure 1C), we used an HSV-1 strain ablated for the vhs gene, which encodes for the viral mRNase (44, 45). Interestingly, the decline of IL6R mRNA still occurred in HSV-1 Δvhs-infected mDCs, but time-delayed when compared to HSV-1 wt (Figures 1B, 6B). The viral protein vhs does not only degrade cellular mRNA, in order to shut off the host protein synthesis, but also negatively affects viral mRNAs, which enables a rapid transition between the three gene expression phases of HSV-1 (70). Furthermore, vhs limits dsRNA accumulation in HSV-1-infected cells (71). The viral protein vhs is active immediately after tegument release and during early stages of infection. Its activity is hampered with ongoing infection via the interaction of the two late proteins VP22 and VP16 with vhs (72, 73). Thus, we suggest that in directly-infected mDCs vhs acts early upon infection to modulate IL6R transcript levels (Figure 6A green line, Figure 6B). Furthermore, the modulation of IL6R surface expression on bystander mDCs was less prominent upon HSV-1 Δvhs infection, compared to HSV-1 wt infection (Figure 6A, blue line). Hence, we conclude that vhs is important for the regulation of IL6R in directly infected mDCs at early time points and more important for IL6R modulation on bystander mDCs. However, based on the described multiple roles of vhs during HSV-1 infection, it cannot be excluded that vhs affects IL6R surface expression in an indirect way.

**Figure 7:**
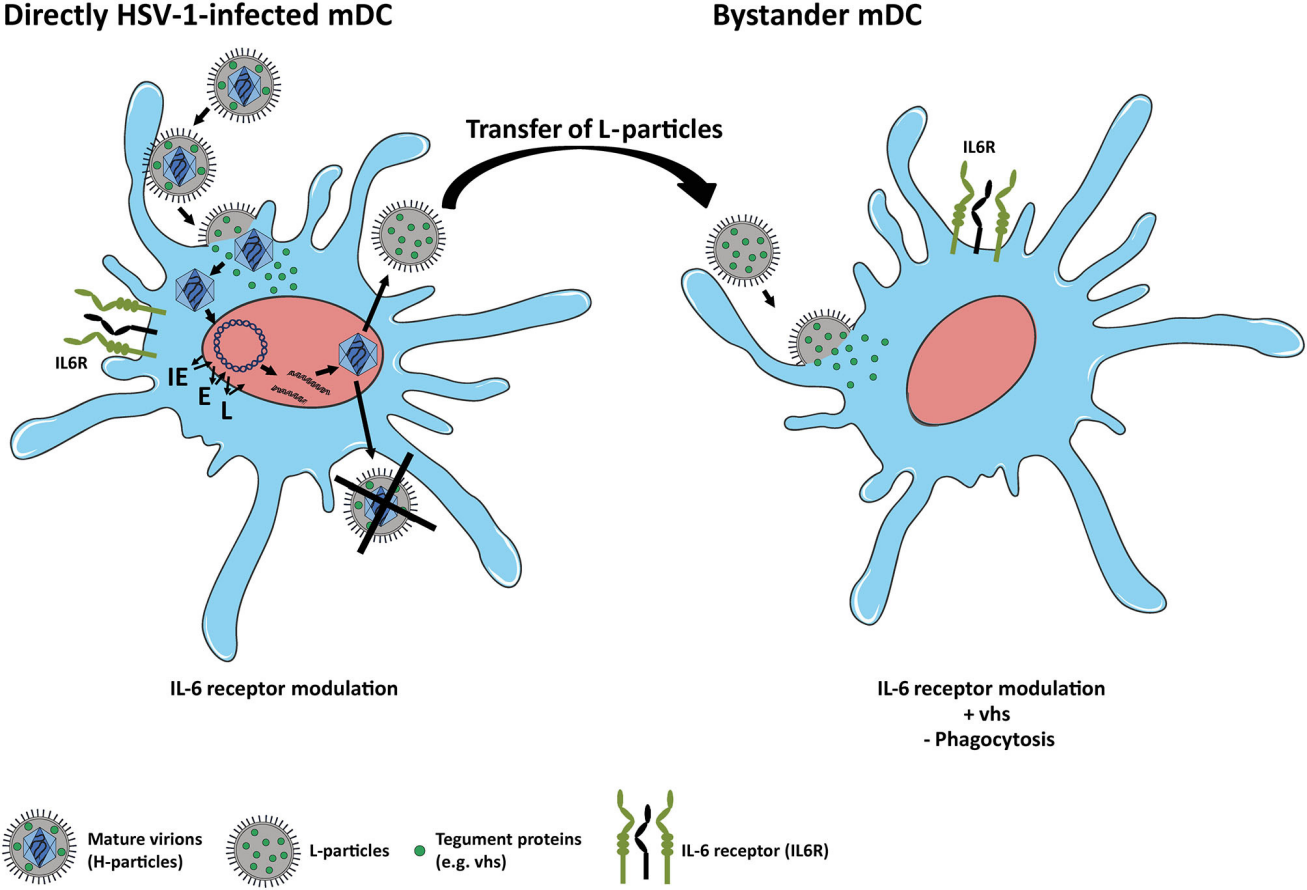
Scheme of IL6R modulation on HSV-1-infected and bystander mDCs. Left: HSV-1 efficiently infects mDCs and modulates IL6R expression on directly-infected mDCs. In mDCs, the production of infectious mature virions (H-particles) is inhibited upon an HSV-1 infection, since newly assembled capsids are trapped within the nucleus. Thus, only capsid-less non-infectious L-particles can be generated by HSV-1-infected mDCs. Right: L-particles are released by HSV-1-infected mDCs and modulate bystander mDCs, e.g., IL6R expression. In this regard, tegument proteins, such as vhs, which are also associated with L-particles, are capable of entering and interfering with bystander cells.

In conclusion, the present study extends recent reports on how HSV-1 affects the expression of distinct proteins in mDCs. In agreement with previous findings on CD83 expression, we here report that IL6R surface expression in mDCs, relative to the mock control, is also reduced not only on directly HSV-1-infected but also on uninfected bystander mDCs. We further provide first experimental evidence that HSV-1-derived L-particles, generated by directly HSV-1-infected mDCs, negatively interfere with IL6R surface expression on mDCs and also account for the loss on bystander mDCs in coculture experiments (Figure 7). We identified the viral-encoded mRNase vhs as inducer of IL6R mRNA degradation and in turn surface protein modulation especially in/on bystander mDCs. We thus hypothesize that HSV-1-derived L-particles contain and transfer important viral proteins, such as vhs (14, 63), to shape the cellular microenvironment. These combined data underscore that L-particles mirror a way to bypass the restriction of complete replication in mDCs and represent a sophisticated strategy of how HSV-1 supports *trans* infection of adjacent cells and hampers the induction of antiviral immune response (14, 15).

## Data Availability Statement

All datasets generated for this study are included in the article/supplementary material.

## Ethics Statement

The studies involving human participants were reviewed and approved by the Ethik-Kommission der Friedrich-Alexander-Universität Erlangen-Nürnberg. The patients/participants provided their written informed consent to participate in this study.

## Author Contributions

The conceptualization of this project was conducted by AB, LP, CH, and AS. Experiments were performed by AB, PM-Z, CD, and CK. Data were validated by AB, LP, CH, AK, and AS. Formal analyses, visualization were performed and original draft was prepared by AB. The manuscript was reviewed and edited by LP, CH, AK, and AS. Supervision and project administration was carried out by LP, CH, and AS. Funding acquisition was performed by AS and LP. All authors contributed to the article and approved the submitted version.

## Conflict of Interest

The authors declare that the research was conducted in the absence of any commercial or financial relationships that could be construed as a potential conflict of interest.

## Abbreviations

BHK: Baby hamster kidney
RT: Room temperature
ICP: Infected cell protein
FCS: Fetal calf serum
EDTA: Ethylendiamintetraacetate
CCR: C-C chemokine receptor type
CXCR: C-X-C chemokine receptor type
CCL: C-C chemokine ligand
CXCL: C-X-C chemokine ligand
CD: Cluster of differentiation
MOI: Multiplicity of infection
MES: 2-(N-morpholino) ethane sulfonic acid
GM-CSF: Granulocyte-macrophage colony-stimulating factor
IL-4: Interleukin-4
EGFP: Enhanced green fluorescent protein
CMV: Cytomegalovirus
LRSC: Leukoreduction system chambers
hpi: Hour(s) post infection
UL: Unique long.
